# Tellurite and Selenite: how can these two oxyanions be chemically different yet so similar in the way they are transformed to their metal forms by bacteria?

**DOI:** 10.1186/s40659-022-00378-2

**Published:** 2022-04-05

**Authors:** Janine Kessi, Raymond J. Turner, Davide Zannoni

**Affiliations:** 1 Until 2018 - Dept of Plant and Microbial Biology, University of Zurich, Zurich, Switzerland; 2 Dept of Biological Sciences, University of Calgary, Calgary, AB Canada; 3 Dept of Pharmacy and Biotechnology, University of Bologna, Bologna, Italy

**Keywords:** Bacterial transport, Bioenergetics, Glutathione, Metalloids, Nanoparticles, Oxyanion reduction, Selenite, Tellurite

## Abstract

This opinion review explores the microbiology of tellurite, TeO_3_^2−^ and selenite, SeO_3_^2−^ oxyanions, two similar Group 16 chalcogen elements, but with slightly different physicochemical properties that lead to intriguing biological differences. Selenium, Se, is a required trace element compared to tellurium, Te, which is not. Here, the challenges around understanding the uptake transport mechanisms of these anions, as reflected in the model organisms used by different groups, are described. This leads to a discussion around how these oxyanions are subsequently reduced to nanomaterials, which mechanistically, has controversies between ideas around the molecule chemistry, chemical reactions involving reduced glutathione and reactive oxygen species (ROS) production along with the bioenergetics at the membrane *versus* the cytoplasm. Of particular interest is the linkage of glutathione and thioredoxin chemistry from the cytoplasm through the membrane electron transport chain (ETC) system/quinones to the periplasm. Throughout the opinion review we identify open and unanswered questions about the microbial physiology under selenite and tellurite exposure. Thus, demonstrating how far we have come, yet the exciting research directions that are still possible. The review is written in a conversational manner from three long-term researchers in the field, through which to play homage to the late Professor Claudio Vásquez.

## Preface

Davide: Janine and Ray, as this contribution is dedicated to the memory of our common friend Claudio Vásquez, it may be appropriate to start our discussion by saying a few words about why and when we started studying the microbiology of metalloids. As for me, it all initiated after reading some of your early papers on the transport and reduction of tellurite and selenite, one of the oxyanion forms of tellurium and selenium. I refer to Janine's work from 1999 [1] and yours, Ray, published the same year in *Microbiology* [2]. These two publications influenced my approach to metalloid biochemistry by suggesting to me the use of facultative phototrophic bacteria to study the bioenergetic aspects of metalloid transport into cells and also to search for tellurite targets in addition to thiols. On the other hand, another publication by Trutko et al. [3] on the oxidation–reduction of tellurite, pushed me to deepen the microbiology of metalloids, which in those years was mainly based on transmission electron microscopy (TEM) analysis of the metal particles seen in various bacterial cell compartments [3, 4].

Ray: As already mentioned by Davide, I started to study tellurite resistance determinants in *E. coli*. I was intrigued in the early 1990’s when the metal ion resistance determinants were being identified, cloned and sequenced, as for those studied at the time, no matter where isolated, a given resistance mechanism proposed was the same for a given metal [5–7]. Yet for tellurite, every clone was unique and distinct from other genes [8, 9].

Janine: I personally started my work about selenite detoxification by microorganisms after reading an article on the adverse effects of selenium, Se, on fish and wetlands receiving agricultural drainage in the San Joachin Valley of California [10]. In those years, in the laboratory of R. Bachofen in Zurich, we were interested to investigate to what extent our bacterial models, particularly phototrophic α-Proteobacteria, were resistant to selenium oxyanions, and whether they could be used in soils detoxification projects.

Davide: Given the aim of this review, I’m pleased to remember the lively conversations I had with Claudio Vásquez in the last period of his research activity on one of the topics, the transport of tellurite in cells, which will be addressed by us, here. These discussions culminated with a short stay in Chile (Dec 2015, La Serena), following an invitation I received from Claudio, to give a talk at the annual meeting of the Chilean Society of Microbiology. This was the occasion during which, not only we spent some time together, but we had the chance to re-examine a few of our results and conclusions. Of course, not all the differences between us were clarified, as also appears in the discussion reported here, but our friendship was further strengthened by the deep empathy that Claudio was able to transmit.

Having said that, before moving on to our discussion, I would suggest to the less experienced in this area of research, to refer to some past and recent reviews that contain general information on the impact of tellurite and selenite in the environment and on living organisms [11–15]. For greater clarity, the topics which will be sequentially addressed in our discussion, arranged as paragraphs and sub-paragraphs, are listed below:

## Introduction

Davide: As anticipated in the preface, most of the literature on tellurite and selenite starts from the strong statement that both oxyanions are highly toxic to bacteria and to higher organisms [8, 9, 11–21].

Ray: Davide, sorry if immediately interrupt you but let’s better explain this concept, first. Even though I have stated this in some of my publications [22–24], it is well established that tellurite is the most toxic of any metal or metalloid to enteric bacteria (from 0.1 to 4 μg/ml) [11, 12], whereas selenite is orders of magnitude less toxic [13, 25]. In addition, there are many microorganisms with very high tolerance that can ‘respire’ these oxyanions [26–32].

Davide: Ray, the claim I made about the extreme toxicity of both oxyanions is an oversimplification and comes from early studies of Anne Summers and Simon Silver in the late seventies [33]. Regardless of this, I’m sure you would agree with me on the fact that numerous reviews have been written on this topic addressing the problem from a strictly microbiological and toxicological point of view, but far less from a biochemical or bioenergetic perspective [13].

Ray: Yes, it’s true. Indeed, a few decades ago many researchers were attracted to the topic of the toxicity of metalloids, even if it was not totally justified given the scarce presence of metalloids in the environment, with the exception of selenium in some areas of the planet [10, 14]. Recently, however, new concerns have been raised from an environmental perspective as the energy production is gradually becoming greener through use of solar cells. Indeed, the decommissioning and disposal of CdTe and CdSe photovoltaic devices results in oxidation and subsequent release of the oxyanions in the environment [34–36].

Janine: Ray, please wait. Before we get too deep into this, I believe we should define more specifically the topic of our conversation/discussion. Thus, I propose to focus on the reduction mechanisms of selenite and tellurite in Gram negative bacteria, mainly, those which are neither assimilatory nor dissimilatory. We know that selenite reduction can be assimilatory leading to seleno-amino acids, particularly the 21^st^ amino acid selenocysteine [25, 37]. Yet dissimilatory reduction also exists for those bacteria that can ‘respire’ using selenium oxyanions as an energy source [38, 39]. In the case of tellurite, the dissimilatory reduction coupled to energy gain is a more recent discovery, but we agree now that it occurs particularly in extreme environments [29, 30, 40, 41]. Regardless, there is no evidence of assimilatory processing of tellurium oxyanions as of yet. However, for both selenite and tellurite, uncoupled reduction certainly occurs, yet it is unclear if it is through collateral metabolism events or focused protection, and we have all explored features of this.

Ray and Davide: Janine, we both agree that we must circumscribe our discussion on the bacterial reduction mechanisms of tellurite and selenite, also because Claudio Vásquez has worked extensively on this research topic. First, however, it is good that we briefly summarize the physicochemical features that distinguish the two oxyanions as well as the differences between elemental tellurium, Te^0^, and elemental selenium, Se^0^.

### On the physicochemical properties of Te^0^, TeO_3_^2−^, Se^0^ and SeO_3_^2−^.

Janine: According to literature, these differences are due to the large difference of electronegativity^1^ between these elements. Indeed, even though both Te and Se belong to Group 16 of the periodic table of the elements, electronegativity values are significantly different: 2.1 for Te, and 2.55 for Se. Another important difference between selenium and tellurium is observed in their respective crystal forms. At ambient pressure and temperature, elemental selenium can exist in its monoclinic or in its trigonal crystalline form. Indeed, EDX (Electron Dispersion X-ray) analysis of bacterial produced selenium particles (SeNP), show narrow peaks at 1.37 keV (SeLα), 11.22 keV (SeKα), and 12.49 keV (SeKβ), indicating that these particles contain pure monoclinic^2^ selenium crystals [1, 28, 49, 50]. Conversely, EDX analysis of bacterial produced tellurium particles (TeNPs), show narrow peaks at 3.769 keV (TeLα1), 4.03 keV (TeLβ1), and 4.31 keV (TeLβ2) together with low intensity peaks at 4.57 keV (Lγ1), and 4.83 keV (Lγ3), indicating that these particles contain pure trigonal tellurium crystals [30, 52]. Based on the current literature (see footnotes 1 and 2), we can then conclude that difference of electronegativity, and therefore of reactivity, as well as difference of crystal structure between elemental tellurium and elemental selenium are the most striking physicochemical differences between these elements and their oxyanions.

Ray: Okay, it’s clear now that the structure of monoclinic Se-crystals together with their instability might explain the observation that Se^0^ nanoparticles produced by bacteria typically show a spherical structure containing the monoclinic crystalline form of selenium [51], whereas rod structures were seen during chemical reduction at temperatures higher than 25 °C [55, 56].

**Fig. 1:**
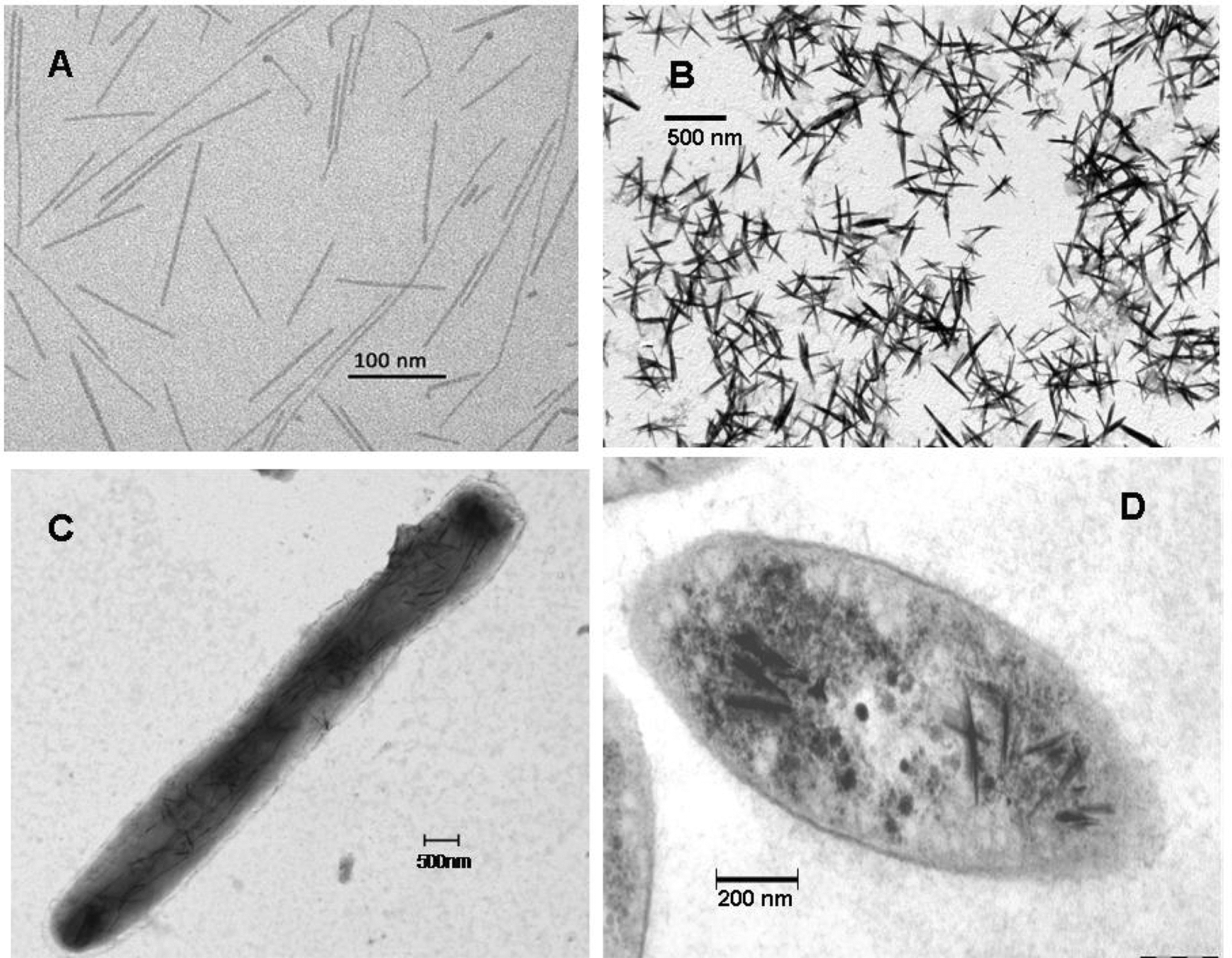
In** A**,** B**,** C** and** D**, TEM images of needle-like Te^0^ crystals generated by chemical reduction (**A**) and in cells (**B**,** C** and** D**) of *Rhodococcus aetherivorans* BCP1 (**C**) and *R. capsulatus* (**B** and** D**), respectively (J. Kessi, D. Zannoni and R.J. Turner, unpublished material)

Janine: At ambient pressure and temperature, in vitro chemical reduction of selenite using glutathione as a reducing agent plus the short-chain phospholipid diheptanoyl-phosphatidylcholine as a detergent, also leads to the formation of spherical, red colored nanoparticles, similar to the bacterially produced Se^0^ nanoparticles. This indicates that monoclinic elemental selenium was formed during the chemical reaction [50]. In contrast, chemical reduction of tellurite using the same procedure, leads to the formation of needle-like elemental tellurium structures, indicating that trigonal elemental tellurium is formed during the reaction (see Fig. 1).

### On the cellular mechanisms leading to the generation of elemental Te^0^ and Se^0^ nanomaterials.

Davide: The differences in physicochemical properties of Te^0^ and Se^0^ remind me the discussions and doubts we had at the beginning of our studies, about differences in redox potentials and speciation forms of TeO_3_^2−^ and SeO_3_^2−^ oxyanions. Indeed, although in most of the early publications dealing with the bacterial response to tellurite and selenite, no attention was given to the actual oxyanion chemical speciation forms interacting with cells, we concluded that tellurite is actually seen as HTeO_3_^−^ at pH 7.0 [57] and this speciation might lead to mimicking the transport of essential anions such as phosphate or sulphate.

Janine: Davide, according to Elrashidi et al. [58] also selenite is partly in the form of HSeO_3_^−^ at pH 7.0. SeO_3_^2−^ is the major species in alkaline soils, whereas HSeO_3_^−^ is predominant in neutral and acid soils. Torres et al. [59] have reported that the HSeO_3_^−^ form is largely predominant between pH 4 and pH 7. This means that cells do not actually see the SeO_3_^2−^ added to the growth medium, but the HSeO_3_^−^ form and this would make selenite similar to tellurite [58, 59].

Davide: Janine, I'm glad you agree with me on this point which has an impact on the mechanism of interaction between oxyanions and cells. As I said at the beginning of our conversation, Trutko’s work [3] pushed me to examine in more detail both chemical and physical properties of metalloids in order to avoid misleading conclusions simply deduced from TEM images of metal particles located closely to cell membranes [3, 4].

Ray: Davide, what exactly do you mean? Are you saying that you don't believe in the possibility that the membrane-bound respiratory oxidases of aerobic Gram-negatives could be involved in the reduction of tellurite to metal Te^0^, as stated by Trutko et al. [3]?

Davide: Yes, I’m afraid so. The electrochemical properties at pH 7.0 of tellurite do not justify its reduction by membrane-bound cytochrome oxidases with high-potential hemes of *aa*_3_ or *cbb*_3_ type [60], that is to say, those present in the aerobically grown species examined by Trutko et al. [3]. Simply put, some conclusions of that *Archives in Microbiology*’s paper [3] were speculations that are not experimentally validated.

Ray: Davide, this is a strong statement but what about the involvement of other membrane oxido/reductases such as nitrate reductases? Verméglio’s lab demonstrated that these enzymes catalyzed selenate, selenite, and tellurite reduction activities [45]. Also, another complex iron sulphur molybdoenzyme (CISM) with selenite reductase activity was recently reported in *Bacillus selenitireducens* [61]. Do you agree on this, at least?

Davide: Yes, I do, but first of all one should remember that in anaerobiosis the oxyanion toxicity values are quite different from those seen under aerobic conditions, and therefore also the reactivity of tellurite and selenite varies enormously. Further, the redox centres of the CISM enzyme have mid-point potentials compatible with the thermodynamics of the oxyanion tellurite in solution at pH 7.0 [57].

Janine: Sorry if I interrupt both of you, but I’d like to mention that Verméglio's group has also shown [62] that although the periplasmic nitrate reductase of *R. sphaeroides f. sp. denitrificans* can reduce tellurite, the V_max_ value for tellurite was 40-fold lower than for nitrate. Accordingly, depletion of the nitrate reductase in *R. sphaeroides* did not modify the MIC of tellurite for this organism [62]. These data therefore indicate a minor role of nitrate reductase in the rate of reduction of tellurite which is in line with what Davide argued.

That said, I notice that you are already focusing on the effects of tellurite on the cytosolic plasma membrane, whereas what is absolutely evident, is that both tellurite and selenite enter the cell cytosol and then are subsequently reduced to their metallic forms, Te^0^ and Se^0^, mainly in this cell compartment. I think we must talk about it before pointing out the redox reactions at the level of the plasma membrane. Concerning the entrance of tellurite to the cells, I read a paper by our friend Claudio Vásquez on the possible involvement of the acetate permease (ActP) in the transport of tellurite in *E. coli* [63]. This agrees with what your group, Davide, observed in *R. capsulatus* [64, 65] and I was amazed, because I thought that in *E. coli*, the tellurite carrier was that of the low-affinity inorganic phosphate (PitA) [66]. Can we then further clarify this aspect?

Ray: Yes, let’s clarify transport first as once reaching the cytoplasm any surviving tellurite or selenite would react with thiol-group containing compounds such as reduced glutathione (GSH) via the classical Painter reaction [67], which was later investigated by Ganther and others [68–70] and further elaborated on by you, Janine [50]. But we have also to consider the path into the cell recognizing possible reactions at different stages of a cell barrier.

Davide: Okay then, let’s move on discussing the transport of these oxyanions (see § 2), then their reduction (see § 3) while remembering that these steps are linked or at least coexisting activities.

## How does selenite and/or tellurite enter bacterial cells?

Davide: Some transporters have been identified to catalyze a tellurite unspecific uptake, such as the low-affinity inorganic phosphate transporter (PitA) in *E. coli* [66], the acetate permease of type 2 (ActP2) in the facultative phototroph *R. capsulatus* [64], and the PstA and PstD proteins (involved in phosphate transport) in the Gram-positive bacterium *Lactococcus lactis* [71]. Specifically, in *E. coli* the low-affinity inorganic phosphate transporter (PitA) was demonstrated to participate in the tellurite uptake of this bacterium, whereas its homologous (81% identity) PitB is not involved [66]. In this research line the Vásquez’s group reported that *pitA* mutation increases up to 4-times the tolerance to tellurite and that the intracellular uptake was decreased by half that of the wild type. They also deleted the *pitB* gene [66], but the mutant exhibited a behaviour similar to that of the wild type, in terms of uptake and sensitivity to tellurite. Conversely, we reported [65] that in *R. capsulatus* the acetate transport system ActP was used for tellurite uptake and that a limitation of its transport (by mutation or by addition of acetate in competition-transport experiments with tellurite) greatly increased the resistance to this oxyanion [72]. Subsequent results by Vásquez’s group aiming to dissect this controversy indicated that in the case of *E. coli*, the ActP transporter(s) showed tellurite uptake activity, but mainly during early exposure to the toxic oxyanion, whereas PitA remained active in longer exposures [63]. This suggests that in *E. coli* the main uptake is always mediated by the PitA transporter. On the other hand, it is important to recall that, when the *actP2* gene from *R. capsulatus* is expressed in wild-type *E. coli* and in *E. coli* Δ*pitA* mutant, the cellular intake of tellurite increases up to four times, suggesting intrinsic structural differences between the ActP of *E. coli* and the ActP2 of *R. capsulatus* [72] (see also Table 1).

**Table 1:** List of transport systems reported to be involved in tellurite- (abbreviated as Te) and selenite- (abbreviated as Se) uptake by cells of various bacterial species (see text for further details and below for symbols and abbreviations used)

Oxyanions	Transport systems	Bacterial species	Method used to define the transport system	Results observed	Refs.
Tellurite	PitA (low-affinity Pi transporter)	*E.coli*	*pitA* gene deletion	*ΔpitA* mutant: increased tolerance to Te	[66]
	ActP (acetate permeases ActP1, ActP2)	*R.capsulatus*	*actP* gene deletion	*ΔactP* mutant: increased resistance to Te	[64, 65]
	ActP2	*R.capsulatus, E.coli*	Expression of ActP2 from *R. capsulatus* into *E.coli*	*E.coli actP2::lacZ* mutant: 4-times higher Te-uptake than w.t	[72]
	ActP	*E.coli*	*actP* gene deletion	^#^Active at short-times of exposure to Te	[63]
	PitA + PitB (low-affinity + putative Pi transporters)	*E.coli*	*pitA* + *pitB* gene deletion	^#^Active at long-times of exposure to Te	[63]
	PstA, PstD (high-affinity Pi transporter)	*L.lactis*	^§^Insertions into *pstA* + *pstD* genes	Strong increase in resistance to Te	[71]
Selenite	Pi transporters	*E.coli*	Addition of Pi to growth medium	Large decrease of Se uptake in Pi amended cultures	[92]
	* Undefined sulphate permease	*E.coli*	Kinetic analysis of sulphate, selenate and Se uptake	Se and selenate compete for a common sulphate carrier	[79]
	PitA (low-affinity Pi transporter)	*E.coli*	*pitA gene* deletion	*ΔpitA* mutant: large decrease of Se-reduction rate	[92]
	PstA (high-affinity Pi transporter)	*E.coli*	*pstA* gene deletion	*ΔpstA* mutant: decrease of Se-reduction rate	[92]
	SmoK protein (ABC transporter like)	*R.sphaeroides*	*smoK* gene deletion	*ΔsmoK* mutant: tenfold increase of MIC for Se	[46]
	Pi transporters	*C.reinhardtii*	Addition of Pi to growth medium	Decrease of Se- uptake in Pi amended cultures	[94]
	Mono-carboxylates transporters	*C.reinhardtii*	Addition of lactate to growth medium	Increase of Se-uptake in lactate amended cultures	[94]

^*^Carrier subsequently identified by others (see [96]) as Sulfate-Thiosulfate SulT permease; #, ‘short- and long-times of exposures’ stand for 5 and 30 min, respectively [63]; §, ‘insertions’ stands for ‘insertional random mutagenesis’ [71]; Se, selenite; Te, tellurite; MIC, Minimum Inhibitory Concentration; Pi, phosphate; PstA, phosphate transport ATP-ase [71]; PastD, phosphate transport permease [71]; ROS, Reactive Oxygen Species; w.t., wild type; Bacterial species: *E. coli, Escherichia coli; L. lactis, Lactococcus lactis; R. capsulatus, Rhodobacter capsulatus; R. sphaeroides, Rhodobacter sphaeroides; Rsp. rubrum, Rhodospirillum rubrum; C. reinhardtii, Chlamidomonas reinhardtii*

Ray: An unfortunate reflection of the literature is a bias of information on Gram negatives versus Gram positives. Gram positives tend to be more resistant to tellurite, yet no adequate explanation has been offered. However, a possible explanation comes from the work of Yu et al. [73] where they demonstrated that surface proteins of *Bacillus subtilis* adsorbs considerable amounts of selenite via the protein’s sulfhydryl sites. Thus, it is not unreasonable to assume that tellurite would get bound up in a similar way leading to less transport and intracellular damage. Alternatively, there may be different paths/rates of transport into the cells of different species.

Janine: Intriguing point, Ray, as the literature demonstrates the complexity in developing uptake experiments involving tellurite and selenite, and therefore the need to carefully examine the data before setting a decisive conclusion. For example, in the paper by Borghese et al. [74], I found it interesting to see that during the first hours of tellurite reduction by *R. capsulatus* cells, many cells die, and this was not only in aerobic cultures, but also in anaerobic photosynthetic cultures grown in a rich medium. Obviously, this proves that tellurite causes serious metabolic damages, most likely at the membrane level since these data were obtained with flow cytometry, a procedure that allows to distinguish between metabolically active and inactive cells, the latter having a permeable membrane. Surprisingly, I did not observe a similar effect in cultures^3^ of *R. capsulatus* B10 grown in the presence of selenite [75]. This result may indicate differences in the selenite resistance mechanism developed by *R. capsulatus* and *Rsp. rubrum*. It must be noted, however, that in both bacterial species the reduction rates were slower than the in vitro rate of chemical reduction of selenite in the presence of GSH (reduced glutathione), which is extremely fast [50]. Considering the high glutathione concentration measured in purple phototrophic bacteria (2–10 mM) [76, 77] one may speculate that the slow rate of cellular reduction could depend on the cells capacity to somehow restrict the entry of selenite, thus raising the level of resistance to this oxyanion.

Davide: Janine, your suggestion is likely right, and it fits with the necessary correlation between the selenite uptake regulation and bacterial resistance to this oxyanion. Early results we published in *Archives of Microbiology* [64] indicated that the toxic effect of tellurite is significantly decreased when tellurite uptake is restricted. Similarly, the early publication of Tomas and Kay [78] and the subsequent work of Claudio Vásquez's group [63] showed that a mutant in the phosphate transport of *E. coli* had a several fold increase of tolerance to tellurite. In this context, it would be interesting to verify whether tellurite uptake is also dependent on the presence of sulfite or selenite because you, Janine, have observed that equal concentration of sulfite and selenite in the culture medium did not decrease the selenite reduction rate in *R. rubrum* cultures; conversely, this activity was strongly inhibited by successive addition of sulfite [75].

Janine: Correct! Thus, the result you are talking about suggests to me that sulfite inhibits selenite reduction at the level of selenite transport. Actually, the idea that sulfur ion transport and selenium ion transport coincide and/or compete is not new, as in the past it was proposed by Lindblow-Kull et al. [79] that sulfate, selenate, and selenite were transported into *E. coli* cells by the same (undefined) carrier, because both selenate and selenite behaved as competitive inhibitors of sulfate uptake. Another work by Lortie et al. [80] in *Pseudomonas stutzeri* reported a significant decrease of the selenite reduction rate in the presence of sulfite. These results are therefore consistent with those published in *Microbiology* [75] indicating a decrease of the selenite reduction rate in cultures of *Rsp. rubrum* amended with sulfite.

Davide: Janine, if I understand your point of view, you would tend to unify the uptake mechanisms of tellurite and selenite, but there is one aspect in the growth curve shown in your paper published in 1999 [1] which is unclear to me, that is: why there is no decrease in the amount of selenite which is present in the culture medium during cell growth, but only after the culture has reached the stationary phase? Can you give an explanation of this behavior in *Rsp. rubrum* which is different from that of *R. capsulatus* despite the fact that these bugs are both facultative photosynthetic bacteria grown under anaerobic phototrophic conditions?

Ray: Davide, before letting Janine answer, let me add something related to this. In the work by Lampis et al. [81] with *Bacillus mycoides*, it is reported that even during the exponential growth phase very little selenite uptake was observed, while at stationary phase rapid uptake and subsequent reduction to Se^0^ was measured. As well as for *Stenotrophomonas maltophilia* isolate SelTE02, the selenite uptake and reduction were correlated because with an increasing concentration of selenite, the uptake and reduction were shifted towards the end of growth and into stationary phase [82]. These observations, Davide, are therefore in line with the result of which you have just asked Janine for an explanation. Furthermore, in the context of this conversation we have ignored the possibility of both selenite and tellurite entering the cell through the sulfate permease system(s). Certainly, selenate does [79] and some selenite is suspected to enter through these systems as well [25, 79]. This makes me wonder how phosphate and sulfate uptake regulation links in, further assuming that phosphate and sulfate uptake are regulated differently in different bacterial species under different conditions and growth phases.

Janine: Ray, I’m afraid we must keep your question open, as no answer is available at the moment. Regarding the question that Davide asked me about the growth curve of *R. rubrum* and the delay in selenite reduction/uptake, I can only say that in line with what you have just mentioned, a similar delay has been observed in other organisms, namely: *Stenotrophomonas maltophilia* [82, 83] *Ralstonia metallidurans* [84], *Rhodopseudomonas palustris* [85], *Staphylococcus aureus* [86] and *Ochrobacterium* sp. MPV1 [87]. In *R. metallidurans*, for example, the selenite reduction kinetics indicated that selenite was significantly transported into the cells only after a long (several hrs) period of exposure of the bacterial culture to the toxic oxyanion [88]. These results suggest to me that bacterial species delaying selenite uptake and reduction may sense the presence of selenite due to its very fast reaction with -SH groups present in outer-cell-membrane proteins, thus producing oxygen radicals (O_2_^−^) [70]. These radicals may elicit a resistance mechanism at the level of the transport system, thus delaying entering of the oxyanion into the cell cytoplasm, and its reduction in this cell compartment. Alternatively, this resistance mechanism could be generated by other ions or molecules formed by the high reactivity of selenite with some other membrane component(s). Unfortunately, this proposal is not yet supported by experimental data and remains hypothetical. I’ll return to this point later. Reading Davide’s report by Borghese et al. [74] showing that part of *R. capsulatus* cells die following their exposure to tellurite, we cannot exclude that this phenomenon also occurs in *R. rubrum* cells exposed to selenite. However, considering the differences of growth and selenite reduction kinetics between *Rsp. rubrum* and *R. capsulatus* [75], one may conclude that the resistance mechanisms they develop may also be different.^4^

Ray: On the other hand, there are other variables to consider, as it has been shown that released metal chelating molecules (siderophores for Fe and more generally ‘metallophores’) are able to bind up, and even reduce selenite and tellurite, at least in the case of *Pseudomonas stutzeri* [90]. This, combined with what could be a significantly high amount of released glutathione (either on purpose or from cell death) which would build up in a closed culture system, would react/complex the oxyanions and decrease their effective concentration. These defence mechanisms against selenite and tellurite oxyanions are very different from the extended lag phase observed with *Rhodococcus aetherivorans* BCP1 [91], which leads to cell adaptation likely through key gene expression changes, as sub-culturing exposed cells remove the lag phase. Regardless, even Claudio Vásquez asked me about this for tellurite several years back, and to us, at that time, it was not clear if this was due to gene expression adaptation or ‘altruistic’ death for community survival.

Janine: Ray, the question you discussed with Claudio is interesting and I wonder whether gene expression adaptation may also occur during the delay of selenite/tellurite uptake and their reduction. I think it would be interesting to determine the gene expression in a few model microorganisms during the time phase between the beginning of the cell growth and the appearance of Te^0^ and Se^0^ precipitates.

### Do tellurite and selenite share the same carriers?

Janine: Considering the various reports about selenite transporters we already mentioned, I’m tempted to conclude that tellurite and selenite should be transported by the same carriers, contrary to what is believed, and that many different transporters are involved in the transport of these oxyanions. In a recent work with *E. coli* [92], for example, it is reported that in cultures amended with 0.1 mM selenite, addition of increasing phosphate concentration up to 30 mM, progressively decreased the incorporation of Se in the biomass, thus indicating that phosphate ions prevented selenite uptake. It must be noted, however, that none of the phosphate concentrations tested, completely suppressed selenite uptake (see also Table 1).

Davide: Janine, let me remind you that the latter competition experiments you mentioned [92], were carried out under experimental conditions very far from the expected 1 to 1 ratio between two reactants that should compete specifically for a common carrier, as vice versa we observed between tellurite and acetate in *R. capsulatus* [64, 65].

Janine: Davide, you are right, but in any case TEM images have shown that the amounts of cytosolic Se^0^ nanoparticles largely decreased with increasing phosphate concentration outside the cells, and that these particles were barely produced in the presence of 30 mM phosphate [92]. Zhu T-T et al. [92] also studied selenite uptake and reduction using mutants of the low-affinity phosphate transport system (*Δpit*A mutant), as well as of the high-affinity phosphate transport system (*Δpst*A mutant) that was investigated by Claudio Vásquez for tellurite transport, some time ago [66]. Interestingly, although both mutants grew well in the presence of 1 mM selenite, its reduction to Se^0^ was slowed down in cultures of the *Δpit*A mutant demonstrating the crucial role of the low affinity phosphate transporter on selenite uptake in *E. coli.* On the other hand, since cytosolic reduction of selenite was not completely suppressed in the *Δpit*A mutant, it is apparent that alternative selenite uptake system(s) are present in *E. coli.* Similar results were reported by Vriens et al. [94] in cultures of the green alga *Chlamydomonas reinhardtii,* together with the observation that addition of lactate to the cultures caused an increase in selenite uptake. It was therefore proposed that lactate activated the monocarboxylate uptake system, and that selenite should also use this system, in addition to the phosphate transporter, to enter the cells of this organism (see also Table 1).

Ray: At this point we agree on the fact that these oxyanions rely, at least partially, on the phosphate transporter to enter the cells and that reduction occurs intracellularly. We should also remember the early work exploring phosphate transport mutants, showing that the outer membrane (OM) porin PhoE provides tellurite resistance [78]. It makes sense to assume that selenite, tellurite, and phosphate movement through the OM entails the same ion porins.

Davide: It is good to mention the fact that the outer membrane is likely involved in the regulation of the oxyanion transport but let me conclude on my thoughts about the acetate permease (ActP). The work of Vriens et al. [94], mentioned by Janine, on the role of the monocarboxylate uptake system in the selenite transport, is quite interesting because it concludes that selenite, like tellurite [64, 65], can enter cells using the carrier for monocarboxylates. In this respect, I’m surprised that Vriens et al. [94] did not recognized the similarities between their results and ours, already present for some time in the literature [64, 65]. Indeed, they could have been the first to propose the hypothesis, which I put forward here, that in certain microorganisms the transport of tellurite and selenite occurs with the same carriers, and that the acetate permease(s) (ActP) in addition to the low-affinity phosphate transporter (PitA), is one of these.

Ray: Certainly, the uptake is a key process that must be further investigated if bacteria are to be used successfully for bioremediation and biotechnological exploitation of these ‘metalloid’ oxyanions. Perhaps this is why Claudio Vásquez, and our friend Vladimir Yurkov, spent time to look for unique organisms with high metalloid oxyanion resistance from extreme environments that could be exploited for such purposes [41, 95]. In Table 1, a list of transport systems reported to be involved in tellurite and selenite uptake by cells of various bacterial species, is reported.

## By what mechanisms and in which cellular compartments are tellurite and selenite reduced to Te^0^ and Se^0^?

### Production of ROS (Reactive Oxygen Species) linked to metalloids reduction

Janine: The hypothesis I have suggested, that the selenite transport indirectly affects both the reduction rate and the cell resistance to this oxyanion, fits with the general view that selenite is reduced to elemental Se^0^ by cytoplasmic GSH (reduced glutathione) as soon as it enters the cell [50]. Further, as the chemical reaction shows that the production of superoxide anions reaches a maximum during the first minutes of the reaction, it’s apparent that ROS (Reactive Oxygen Species) are rapidly produced in the cytoplasm. Considering the work of Borsetti et al. [97] together with that of Chasteen et al. [98], I believe that tellurite reduction would follow the same reduction mechanism. On the other hand, the results of Davide’s group that tellurite can also be reduced to Te^0^ in the periplasmic space, along with the negative effects of mutations of the cytochrome *c* maturation (Ccm) system on tellurite reduction rate and efficiency [99], suggest that a more detailed analysis of the different reactions taking place in this cell compartment, is needed.

Ray: The observation of ROS production from oxyanion exposure that different research groups report, is confusing. I do not doubt that different people have seen this phenomenon; however, one must consider that ROS are produced both directly in defined chemical reactions via Fenton and Haber–Weiss type reactions, or indirectly through a decoupled electron transport chain releasing electrons [24]. This is why many biocides lead to ROS production as the membrane integrity is lost, leaving cytochromes and quinone radicals unable to efficiently target their electrons through the electron transport chain (ETC), thus they end up passing their electrons on to oxygen or water or even sulfur or nitrogen groups producing radicals. For this reason, one sees misinterpretation in the literature where various stressors are defined to produce ROS directly, but cause and effect are difficult to define, particularly with the ROS sensing dyes. For example, in 2009, we evaluated different metal(loids) comparing ROS production *vs* R-SH (reduced thiols) oxidation [23]. It was clear that in *E. coli*, selenite and tellurite oxidizes cell R-SH; however, only selenite produced ROS but not tellurite. In fact, selenite led to increase gene expression in *sodA, soxS*, *oxyR* and *rpoS* (the systems that respond to oxidative stress), whereas tellurite only led to a moderate increase in *oxyR*. My interpretation for this difference was through the pathway of selenite to selenocysteine will lead to selenides and ROS [25], whereas tellurite may not follow the same reaction pathway(s), or the latter may be kinetically much slower, producing tellurocysteine at lower levels, thus less ROS. However, if my conjecture is sound in *E. coli*, then I have no explanation why, in other studies, higher ROS production was observed in the presence of tellurite.

Janine: Ray, as already stated, tellurite is significantly more reactive than selenite and, unlike selenite, was shown to be included into proteins very rapidly in their post-translational state [100] generating tellurides or other tellurium derivatives. Since diaryl ditellurides and diorganyl tellurides were shown to exert thiol peroxidase activity [101, 102], we can expect that this reaction may occur so to inhibit the induction of enzymes involved in peroxidase activities. Such an effect could possibly explain the moderate induction of enzymes involved in ROS degradation by *P. pseudoalcaligenes* KF707 in the presence of tellurite [103]. This reasoning, however, contrasts with other results reported by Tremaroli et al. [103], indicating that bacterial cultures pre-treated with ROS generators such as paraquat and diamide increased tolerance of KF707 cells to tellurite.

Davide: Janine, I’m glad you mention our early results in KF707 cells so I can explain them in the light of what we know now on tellurite reduction by this bacterial species. In this aerobic and polychlorinated biphenyl degrading bacterium, tellurite triggers an increase in ROS production, but SOD enzyme activity was far more stimulated by addition of oxidant agents such as paraquat and diamide than it was by tellurite addition. At that time, we concluded that the mild SOD stimulation was probably due to the very slow kinetics of both tellurite uptake and ROS release [103]. Indeed, almost ten years later [72] we demonstrated that *P. pseudoalcaligenes* KF707 belongs to the group of bacteria that contain a type 1 acetate permease (ActP1), whose tellurite transport activity is up to 100 times lower than that catalyzed by ActP2, which is present in *R. capsulatus* [72]. Therefore, the uptake of tellurite into KF707 is so slow that in vitro kinetic experiments in a short time scale (> 10 < 60 min) did not reveal any consistent SOD activation [72].

Ray: So, following from earlier, we agreed that transport uptake rate has a relation to oxyanion reduction rate and culture growth phase. From what you say then, the rate of these processes dictates whether the oxidative stress response system in the cells will be overwhelmed and thus the ROS levels observed. But is there still more to it?

Davide: Ray, I would say yes! Back to your comment about ROS production by dysfunction of the ETC, as particularly observed from complex I or NDH-I [104], is worth mentioning a paper published in 2015 by Vásquez’s group [105]. This publication shows a working scheme in which the *bd*-I terminal oxidase is proposed to use tellurite as alternative electron acceptor and also that the NDH-II works as an NADH dependent tellurite reductase [106]. Further, since both NDH-II and *bd*-I redox enzymes catalyze reactions which leave the 4 reduction-process of an intermediate Te(II) species unstable, generation of ROS was proposed. It is needless to recall in this context, that I disagree with the conclusions suggested by this latter work for the same reasons I have given in my previous interventions to criticize the work of Trutko et al. [3]. Vásquez’s group also suggested that there is a switch to anaerobic based metabolism under tellurite exposure, which would also lead to less ROS [107]. In my opinion, a simple explanation for these different observations about ROS production could be the differences in growth modes and the metabolic cell capacity to select optimal energetic growth conditions. On the other hand, the misconception that tellurite may be an alternative acceptor to oxygen for various respiratory oxidases [3], has contributed to confusion. Tellurite can undoubtedly accept electrons along the electron transport chain (ETC), but the interaction likely occurs at the level of the quinone pool which functions both as a donor for quinol oxidases and as an acceptor in NADH-dependent quinone reductases.

### On the role of periplasmic and cytosolic GSH in the reduction of metalloids

Ray: Davide, I agree with your strong criticism on the fact that tellurite is unlikely an alternative acceptor to oxygen in the oxidases of the ETC, but let’s return to Janine’s question on the presence of GSH in the periplasmic space which would implicate the generation of Te^0^ and Se^0^. I have no doubts, Janine, that there is GSH in the periplasm, because others have established it [108, 109]; further, the presence of carriers for glutathione in the plasma membrane also argue in favour of this evidence [108, 110]. However, I am sure we all agree that the cells have much lower quantities of GSH in the periplasm than in the cytoplasm. I can accept the idea that glutathione is in disequilibrium between these compartments, but it is difficult to explain why GSH, which is one of the major molecules responsible for maintaining the cell-redox state, should be consistently present in a compartment which is in direct contact with the extracellular environment, and thus would be oxidizing, leading to all the GSH to be GSSG. It may be that mine is a dogmatic vision, but the literature data does not prove to me otherwise [110].

Janine: Ray, I fully understand your point of view but according to Smirnova et al. [111] reduced glutathione undergoes continuous transmembrane cycling between the cell periplasm and growth medium. Further, I find it interesting that in *Ralstonia metallidurans* and in *Synechocystis sp.* PCCC 6803, Sarret et al. [88] and Gouget et al. [112] demonstrated by X-ray absorption near edge structure (XANES) spectroscopy the formation of organoselenium (R-Se-R) immediately after the addition of selenite to the cell culture and in a longer time the formation of selenodiglutathione (S-Se-S). These two reports tend to suggest that selenite, when added to the culture medium, rapidly reacts with outer-cell-membrane components, thus leading to the formation of R-Se-R compounds. Conversely, the slower kinetics linked to the initial formation of selenodiglutathione (S-Se-S), and then of Se^0^, would occur in the periplasm where GSH is present in low concentration.

Davide: Janine, it is true that the report of Smirnova et al. [111] is puzzling, but I wonder if the presence of glutathione in the growth medium of batch cultures might simply be due to cell lysis. Further, as the cell periplasm is rich in biosynthetic pathways based on redox processes where glutathione plays a fundamental role [110, 113, 114], its concentration (in the micromolar range) must necessarily be under strict control by the cell [110]. Despite the experimental uncertainties, I am convinced that all the data mentioned so far lead us to conclude that the toxicity of oxyanions mainly comes from their reactivity in the cytoplasm and not outside the cytoplasm. It follows, that as long as tellurite or selenite are on the outside, and by outside, I mean also in the periplasm, their toxicity is minimal. In our experience, whenever we blocked tellurite externally, i.e., in *R. capsulatus* ActP2 minus mutants, the toxicity markedly decreased [65]. This of course does not mean that tellurite cannot be reduced in the periplasm by any GSH present and that it can also generate oxygen radicals there, but their quantities are not comparable to those produced in the cytoplasm. Therefore, my point of view is that, if GSH is involved in the reduction of periplasmically located tellurite, the toxic effects due to free oxygen species are minimal compared to those observed in the cytosol.

Janine: Davide, I completely agree with you. Consistently, as glutathione concentration is very low in the periplasm compared to that of the cytoplasm, the redox potential of the periplasm is significantly higher compared to that of the cytoplasm (− 165 mV and − 260/− 280 mV, respectively). Consequently, glutathione is mostly in its oxidized diglutathione form in the periplasmic cell compartment. However, considering the oxidative stress generated in cells faced to tellurite or selenite, we have to consider that these cells are subjected to numerous metabolic changes, such as disruption of the cell redox equilibrium, together with that of glutathione concentration between cytoplasm and periplasm [115], with consequences on the cytochrome biogenesis and repair [108, 116]. Davide, I remind you that the presence of low amounts of Te^0^ and Se^0^ in the periplasm cannot be denied and in fact you’ve shown that the disulfide binding proteins, DsbA and DsbB, are somehow involved in periplasmic tellurite reduction [117].

### Can tellurite be reduced in the periplasm through the use of reducing power from the cytosol?

Davide: Janine your observation is correct and indeed the membrane-attached disulfide binding protein, DsbB, is able to funnel the reducing power from the reduced ubiquinone (UQH_2_) pool to tellurite in *R. capsulatus*. However, I’d like to point out that the results you mention were obtained in isolated membrane fragments [117]. Therefore, it’s hard to establish how much this electron transport pathway contributes to tellurite reduction in intact cells. As we have shown, the effect of adding tellurite (oxidant) to membranes in vitro with the UQ pool in a pre-reduced state, induces an unexpected—rapid and full—reduction of the membrane cytochromes of *c*-type. The reducing power to both tellurite and cyt *c* derive from the activity of the UQH_2_→DsbB pathway as the same experiment done with membranes of a DsbB deficient mutant of *R. capsulatus*, do not show any reduction of the *c*-type cyts [117]. It is therefore concluded that the membrane-attached DsbB is required to connect the membrane UQH_2_ pool with the exogenously added tellurite. Further, if this were true even in intact cells, it would follow that periplasmic tellurite can be reduced by cytosolic reducing agents funnelling electrons into the electron transport chain.

Janine: Davide, although your experiment was quite elegant and tricky, I think we also must consider, here, that superoxide anions were shown to reduce cyt *c* [118]. As for the formation of superoxide anions during bacterial reduction of tellurite or selenite, it is likely that analysis of the electron flow, when these reactions take place, will be complex.

Davide: Janine, in theory the possibility that superoxide anions can reduce cyt *c* in solution cannot be excluded, but in our case [117], the tellurite-induced reduction of the membrane bound cyts *c* was inhibited by antimycin, which is a specific inhibitor of the UQ:cyt *c* oxidoreductase (or cyt *bc*_1_ complex). Therefore, the involvement of ROS generated by tellurite reduction is unlikely. I underline that our observation is similar to the experiment reported by Wikström and Berden [119] called "oxidant-induced cyt *b* reduction" that is a thermodynamic paradox which can however be explained by the Q-cycle mechanism itself. Thus, from the theoretical and practical point of view, the DsbA/DsbB couple can connect the membrane redox chain with exogenous electron acceptors/donors that are in redox equilibrium with each other.

Janine: The role of DsbA/DsB is now clear to me, yet you’ve also shown a role for Ccm (Cytochrome *c* maturation) system in the periplasmic reduction of tellurite, not only in membrane chromatophores, but also in intact cells [99]. How do you comment on this?

Davide: First of all, it is important to say that all the soluble components which are lost in isolated membrane fragments, are present in whole cells. Therefore, in this latter case I would be less definitive in terms of conclusions although the most significant phenomenon we observed in intact cells, was the inhibitory effect of tellurite on the biosynthesis of soluble cyt *c*_2_ and much less on the membrane linked cyt *c*_y_ [99].

Janine: In my opinion, this difference might be due to the different location of these two *c*-type cytochromes. The heme group of cyt *c*_y_, which is a plasma membrane-bound cytochrome, could be shielded from the ROS produced during cytosolic or periplasmic tellurite reduction, whereas the cyt *c*_2_, which is a soluble periplasmic cytochrome, might have its [Fe-S] cluster more exposed to ROS, which degrade [Fe-S] clusters and suppress the cyt *c*_2_ metabolic function ([120] and references therein). Alternatively, the inhibitory effect of tellurite on the cyt *c*_2_ may derive from cytosolic H_2_O_2_. Indeed, bacterial membranes are permeable to H_2_O_2_ [121] and heme prosthetic groups are damaged by hydrogen peroxide [122]. Then, degradation of cyt *c*_2_ in the periplasm might depend on H_2_O_2_ produced in high amount in the cytoplasm and having passed through the membrane.

Davide: Janine, aside from the fact that your proposal(s) requires experimental verification, there is no data to suggest that the two hemes are otherwise accessible to ROS or hydrogen peroxide. What is certain, is that cyt *c*_y_ and cyt *c*_2_ are both located in the periplasm and that they both participate in the photosynthetic and respiratory electron flow [123]. Further, tellurite and *c*-type hemes do not see each other thermodynamically, but they only do so via redox mediators. As far as I know from the papers published by Fevzi Daldal’s group [124, 125] heme *c*_2_ and heme *c*_y_ have similar redox potentials but they only differ in their holo-forms, as cyt *c*_y_ is attached to the membrane with a protein anchor of variable length which ranges from 42 to 68 amino acids [125]. Unfortunately, we know very little about the biosynthetic pathway of cyt *c*_y_ and, therefore, I do not want to venture myself into considerations in this regard [126]. In my opinion, the effect of tellurite is not due to differences in location or structure of cyt *c*_2_ and cyt *c*_y_ and, as far as I know, the ROS production in the periplasm of *R. capsulatus* is minimal [97]. Thus, I would be inclined to believe that the effect of tellurite on the amount of cytochromes *c* is an indirect effect via the redox imbalance of the Ccm system [99].

Janine: I agree that the ROS production in the periplasm may be much lower compared with that produced in the cytoplasm. This is also consistent with the fact that reduction of tellurite or selenite in the periplasm should be very slow compared with the reaction occurring in the cytoplasm. Such a difference can be explained by considering the difference of GSH concentration together with the difference of redox potential between these two cell compartments, i.e., bacteria contain millimolar levels of GSH in their cytoplasm [76, 77] whereas only micromolar concentrations are present in the periplasm [127]; accordingly, as we already claimed earlier, the redox potential is largely more oxidizing in the periplasm compared with that of the cytoplasm [128].

Davide: Janine, I’m glad you agree with me and Ray that the periplasmic reduced / oxidized glutathione ratio is too low for ensuring a strong electronegative potential. So, to summarize, if in Gram negatives the cytosol, the periplasm, and the external space are three different compartments separated from each other by membranes with the same chemical-physical characteristics, the three compartments are likely to maintain a redox imbalance between them despite being connected in various ways. Indeed, the periplasmic pH of growing cells would be acidic (as much as two pH units lower than cytosolic pH) due to proton extrusion. These localized pH’s micro-domains affect the equilibrium of the different forms of tellurium, shifting to more positive values the potentials of the redox couples involved. Keeping these basic concepts in mind, it is not possible to accurately predict the interaction of tellurite with specific respiratory redox complexes, although the most likely thermodynamic interaction would be at the quinone pool level (*E*m,7 of the redox couples Q^·−^/Q and Q/QH_2_ of − 200 and + 90 mV, respectively) as proposed by Borsetti et al. [117].

Ray: Thus, in line with our early paper [117] and with what you just said about the role of DsbB in taking electrons from reduced quinone, it makes sense to conclude that tellurite is capable of extracting electrons from the Q/QH_2_ pool and, consequently, be reduced to Te^0^. If so, then tellurite would harm the creation of the proton electrochemical gradient (ΔμH^+^) used for ATP production. This could explain our observation by NMR, that ATP levels are rapidly depleted in the cytoplasm as well as a loss of the pH gradient across the membrane [22].

Davide: Correct. In my opinion, the effect of tellurite is twofold, that is: it interferes on the membrane electron-transport chain at the Q/QH_2_ pool level, so to affect the formation of the membrane potential and it further decreases the potential itself by using it for the mechanism of entry into cells which is ΔpH dependent [13]. By doing so, a futile cycle is triggered which tries to counterbalance the drastic decrease of the ΔμH^+^ by raising up the ATPase activity to recover the electrochemical gradient. As a consequence, the endogenous pool of ATP is rapidly hydrolyzed. This is what you have seen by nuclear magnetic resonance [22].

Janine: Ray and Davide, I’m sure you realize that in this context the regulation of glutathione concentration between cytoplasm and periplasm becomes crucial. Indeed, according to Holyoake et al. [115], the glutathione/cysteine exporter CydDC is a heterodimeric ATP-binding cassette contributing to the maintenance of redox homeostasis, and this function has an intricate relationship with cellular metabolism.

### On the multiple roles of GSH and on multiple reactions that lead to the reduction of tellurite and selenite to Te^0^ and Se^0^.

Janine: Davide, remaining on the subject of redox homeostasis, perhaps your results indicating the role of the DsbA/DsbB/Ccm system complex in the delivery of electrons to tellurite and consequent alteration of the biosynthesis of cyt *c*, can instead be traced back to the multiple roles of GSH. As far as I know, glutathione has emerged as a post-translational regulator of protein function under conditions of oxidative stress. In a paper by Masip et al. [129], it is reported that glutathione is not only directly involved in the reduction of selenite/tellurite, but also in the control of other functions in cells under oxidative stress. Many different metabolic roles of glutathione are reported in the literature such as: non-toxic reserve of cysteine, regulation of the redox state, adaptation to various environmental stresses such as oxidative stress, temperature stress, osmotic stress [130]. Additionally, glutathione has been shown to be involved in protein folding as a molecular chaperone [131], in maintaining the cell surface thiols in a reduced state [127], in holo cytochrome assembly [132, 133]. We have also to keep in mind, that the high concentration of GSH in the cytoplasm, being 500–1000 times higher than that of other intracellular redox systems, such as NAD(P)H, makes this compound the most important cell redox buffer [130].

Ray: Yes, there are considerably different concentrations of GSH in the different compartments [111] and I certainly agree that GSH has indeed been linked to multiple cellular functions. On the other hand, we cannot leave out the additional thiol redox mediators of thioredoxin and glutaredoxin [134] which are prevalent cytosolic proteins that increase the effective RSH concentration for redox stasis. It is worth mentioning then, that these compounds convey reducing equivalents from NAD(P)H to glutathione and thus to tellurite and selenite. What always surprised me, is when we have measured total RSH levels in *E. coli* during metalloid exposure, we saw an exponential loss in the presence of tellurite. In the case of selenite, we also saw this rapid decrease in RSH, but this was soon followed by a recovery [135]. This always had implied to me that there was quite a difference in the reactivity and thiol redox response between these two metalloids, which was independent of transport and ROS response. In fact, we showed that addition of selenite would actually protect the cells against the more sever tellurite toxicity [48]. How does this rationalize with what you are saying?

Janine: In my view, the protection of cells by selenite against the more severe tellurite toxicity might suggest that the cell defence system put in place in the presence of selenite is also effective against tellurite. Unfortunately, this “defence-system hypothesis” is not currently supported by molecular evidence. About the much higher tellurite toxicity, compared to that of selenite, I recall the work by Garberg et al. [100] showing that tellurite can be incorporated into proteins and that this process leads to a strong decrease of the glutathione peroxidase activity. Since the glutathione peroxidase has SH- groups at its active centre, these groups are likely targets for tellurite. Conversely, according to Garberg et al. [100] selenium is not post-translationally inserted into proteins so that its toxicity is primarily due to the formation of ROS and a drop of cytosolic GSH.

Ray: I think this observation is explained by the difference between the stability of the intermediates of thiol reactions. RS-Te-SR appears to be quite stable, whereas RS-Se-SR can be quickly reduced with further RSH equivalents to RSSR and Se^0^. Glutathione reductase accepts RS-Se-SR as a substrate, but not RS-Te-SR (Turner’s group, unpublished). Thus, tellurium becomes captured in a protein with vicinal thiol groups, whereas selenium is released.

Davide: Regarding the cellular defence system against these two oxyanions, what I know about it, is that the capacity of moving reducing equivalents from the cytosol to the periplasm through the use of the disulphide-binding proteins, DsbA/DsbB, requires a cytosolic machinery powered by thioredoxins [136, 137].

Janine: Yes, it’s true but we have to keep in mind that GSH was shown to play a predominant role in the regulation of the redox system, maintaining the redox potential in both cytoplasm and periplasm. As I mentioned before, the high concentration of glutathione makes it a crucial component for the maintenance of the cell redox buffer [130].

Ray: Janine your statement is correct, but we can’t ignore the role of cytoplasmic ‘moonlighting’ enzymes, as for example Se and Te oxyanion reductases. Such reactions in the cytoplasm do not require GSH as they take electrons from NAD(P)H directly. This would be different than the NADH reductases feeding electrons through the various electron transport chain components, leading to oxyanion reduction at the membrane level [3, 105] and it is also different from nitrate reductases [45]. For tellurite reduction in the cytoplasm, Claudio Vásquez has reported several activities beyond the electron transport chain. He first concluded that catalases were the NADPH-dependent tellurite reductase [138]. Vásquez’s group found several other enzymes with tellurite reductase activity over the years including: isocitrate- and 6-phosphoguconate-dehydrogenases [139, 140], dihydrolipoamide dehydrogenase [141, 142], thioredoxin- and alkyl hydroperoxide-reductases, flavorubredoxin reductase [143] and glutathione reductase [86, 144]. Most of these use NAD(P)H as the source of electrons, but also several are paired thiol chemistry enzymes. Claudio also postulated that other enzymes with this combination likely exist, that can catalyze this activity leading to Te nanostructure accumulations [145], independently of the type of electron transport chain activity present in the organism.

Janine: Ray, I’m glad that you remembered these contributions from Vásquez's research group which essentially concern the fate of cytosolic tellurite. As for the enzymatic selenite reduction to elemental selenium, two mechanisms have been proposed in the past, namely: a periplasmic dissimilatory nitrite reductase [146], and an inducible sulfite reductase [147]. However, according to my results [75], addition of nitrite or sulfite together with selenite in the growth medium of *Rsp. rubrum* and *R. capsulatus*, modified the growth kinetic as well as the kinetic of selenite reduction, when compared with cultures amended with selenite only. But even so, in cultures of *Rsp. rubrum* as well as in cultures of *R. capsulatus,* selenite was reduced to elemental selenium not only when added to the culture medium at the beginning of growth, but also when added after the end of the exponential growth phase. Conversely, nitrite and sulfite were reduced only when added at the beginning of growth. Considering the fast reaction of tellurite (or selenite) with -SH groups, it is apparent that tellurite can be reduced by paired thiol enzymes, and that these enzymes participate in tellurite reduction. However, considering the very fast reaction of tellurite (or selenite) with GSH, along with its millimolar concentration in the cytoplasm, it is also clear that the reduction with GSH is largely predominant compared with that occurring with other -SH-containing molecules or more generally with enzymes present in much lower concentration than GSH in the cytoplasm. I’m also convinced that the main role of both thioredoxin- and glutathione-reductases is to regenerate -SH groups of various metabolites, and in particular, to reduce glutathione in order to restore the redox state of the cells.

Davide: Ray and Janine, I agree with you that a great deal of literature data demonstrates the broad spectrum of reactions involving the reactivity of tellurite and selenite with different cytoplasmic enzymes. However, there is another aspect that confuses me even more. That is, despite the historical fundamental observation that tellurite-tolerant bacteria transform TeO_3_^2−^ into elemental Te^0^, which is assumed to be no longer toxic to cells, the same occurs when tellurite-sensitive bacteria are grown in the presence of sub-lethal toxicant concentrations [42, 57, 74]. In addition, Yurkov et al. [148] observed tellurite resistance without oxyanion reduction in some species of obligately aerobic photosynthetic bacteria, i.e., *Roseobacter thiosuphatophilus*, suggesting that tellurite tolerance does not necessarily depend on the formation of Te^0^ precipitates. Yet, given that many of the systems we are discussing here likely exist in these isolates, why is there no Te^0^ observed?

Janine: With sub-lethal as well as in the presence of higher toxicant concentrations, tellurite/selenite generate various tellurite/selenite derivatives with -SH-containing metabolites. However, according to the work by Garberg et al. [100], incorporation of Te in cellular components was shown to be much higher compared to that measured for Se. Consequently, it is possible that in the presence of low tellurite concentrations such reactions might immobilize the whole amount of Te present in the cells.

Davide: Janine, your proposal would be likely if the MIC (Minimum Inhibitory Concentration) values ​​observed by Yurkov et al. [148] were 100 or 200 times lower than those reported, i.e. 1–2.5 mg/ml. In this particular case, the answer to the question I posed must be sought on other resistance mechanisms, namely: i) tellurite methylation, with generation of volatile derivatives as dimethyl telluride (CH_3_TeCH_3_) and dimethyl di-telluride (CH_3_TeTeCH_3_) [149], ii) the transfer out of the cells of tellurite using anion transport systems such as the arsenical ATPase efflux pump [25], and iii) Te^0^ nanocrystals cell extrusion through the formation of either OM (outer membrane) vesicles or porin-mediated transport, as both response mechanisms to metalloid-induced oxidative stress. It should also be underlined that obligately aerobic photosynthetic bacteria are peculiar microorganisms as they are unable to carry out photosynthesis in anaerobiosis [150], which is due to the lack of a tight control of the growth redox conditions necessary for an optimal photophosphorylation activity (Eh > 80 mV < 140 mV) [151]. Hence, their need to grow aerobically while having the additional possibility of carrying out photosynthesis during the daytime hours in surface/aerated waters. In such a habitat, therefore, mechanisms of photooxidation or methylation of tellurite should prevail to protect the bacteria from the high toxicity of the oxyanion. Further, I would not overlook an interesting result reported in Yurkov et al. [148], which unfortunately has not been commented on by the authors themselves, that is the presence of dozens of vesicles of variable size between 20 and 50 nm closely attached to the outer membrane of *Roseococcus thiosulphatophilus*. This peculiarity, that I’m sure Janine will like very much, introduces us to the next topic in which we’ll discuss, among other things, the fate of the elemental Te^0^ and Se^0^ particles produced in the cytoplasm.

## What is the fate of the elemental Te and Se particles produced in the cytoplasm and those produced outside the cells?

Janine: Yes, now this brings me to a question that has been on my mind: considering the strong capacity of several bacteria to reduce tellurite and selenite to elemental Te^0^ and Se^0^, may I ask to Davide why the idea of adding an artificial electron carrier such as the naphthoquinone—lawsone—to generate Te^0^ nanoparticles (TeNPs) outside the cells of *R. capsulatus* [156, 157] rather than naturally, inside?

Davide: Janine, we explored this procedure for curiosity but also towards biotechnological applications. The addition of an artificial electron carrier such as lawsone (also known as hennotannic acid or henné), shifts the equilibrium of the reaction between the Q/QH_2_ pool (lipophilic) and tellurite (hydrophilic), from the membrane phase to the aqueous phase. We found that not only lawsone (*E*_h_^0’^ = − 0.145 V) was shown to be a perfect redox mediator,^5^ but we also found that when added in catalytic (micromolar) quantities, it was not metabolized by the cells [157]. Apparently, with high amounts (millimolar) of substrates such as pyruvate or malate, lawsone is not an attractive and alternative carbon source for cells. For us, this was an important observation because, from a biotechnological point of view, high quantities of TeNPs could be produced outside the cells without having to constantly add lawsone to the growth medium [157]. In this way, the TeNPs in the form of needle-like black crystals can easily be isolated by simple filtration (see also footnote 5).

Janine: So, if I understand correctly, the purpose of this approach is to isolate the metal particles more easily when applied to environmental detoxification procedures especially in an aquatic habitat. However, the problem remains that the particles are nevertheless largely contaminated/surrounded by organic material. Further, it is important to note that several signals detected by mass spectroscopy of SeNPs produced by *Rsp. rubrum* and *R. capsulatus* correspond to various metabolites of the photosynthetic apparatus along with natural phospholipids, suggesting that these particles were coated by components of the intracytoplasmic membrane system [120].

Davide: Yes, I know that even the nanoparticles produced in the presence of lawsone are ‘contaminated’ by an organic coating rich in proteins, including porins [158]. However, this is not a problem but more of a benefit. Indeed, the organic components surrounding both TeNPs and SeNPs allow them to remain stable in aqueous suspension so as to be easily and quickly separated from the cells. Conversely, if the nanoparticles were only synthesized inside the cells, the time and cost of obtaining the NPs would be unsustainable due to the need to break the cells with mechanical treatments, and the subsequent separation of the NPs from the cellular fraction. Using the lawsone, on the other hand, once the particles in the suspension medium have been obtained, the organic fraction can be stripped off if deemed necessary for the application.^6^

Janine: Davide, your report on the presence of porins associated with the nanomaterial coat is quite interesting [158], but not surprising because I also observed the presence of porins in the organic coating of selenium particles (Kessi’s group—unpublished results). This introduces another intriguing topic, that concerns not only the fate of nanoparticles produced in the cytoplasm but also their mechanism of biosynthesis.

Ray: The presence of proteins and lipids does not surprise me. Together with the Vallini and Lampis‘ group at the University of Verona (Italy), we have evaluated the presence of lipid coat to the SeNPs from *S. maltophilia* and *Ochrobactrum sp.* MPV1 [159]. We followed this up exploring the coatings of both intra- and extra-cellular SeNPs from a variety of environmental isolates [160]. From this, we saw a mixture of lipids, proteins, and carbohydrates (exopolysaccharides; EPS) with lipids typically at the majority. Unfortunately, to date, few have looked closely at the full composition of molecules associated with biogenic nanomaterial, and I am sure there will be some surprises such as the presence of cytosolic metabolites associated with the coating. Regardless of this, there is enough evidence to indicate that such biomolecules coating the nanomaterial provide high thermodynamic stability to NPs [161], compared to chemically produced NPs. In fact, our recent data suggests the different physiological states of a bacteria could lead to different organic components associated with the NP cap that likely participate in tuning the size and shape of the particles [56].

Janine: I also agree that cytosolic metabolites participate in the formation of particle coating. In this respect, various components of the photosynthetic apparatus were reported to be associated with SeNPs generated by both *Rsp. rubrum* and *R. capsulatus* cells [120]. On the other hand, something else bothers me. Davide, comparing the images shown in Borghese et al. [156] with the pictures I obtained in *Rsp. rubrum* during the selenite reduction [1], as well as in the unpublished pictures reported here, it clearly appears that in all cases selenite was reduced in the cell cytoplasm. In my report [1], selenite particles were seen in the cell cytoplasm during reduction i.e., particles were seen intracellularly only, during the whole reduction time. However, two days after the reduction was complete, most particles were seen in the culture medium [1]. The same result was obtained using cell centrifugation in a sucrose step gradient, this experiment demonstrating that the buoyant density of the cells increased in the presence of selenite during the reduction phase and reverted to the buoyant density of control cells after the reduction was complete [1]. It was seen that a few particles are present on the cell surfaces three days after the beginning of growth which corresponds to the end of the reduction time [1]. According to these results, no exogenous electron carrier such as the lawsone used by your group, Davide, in *R. capsulatus* cultures is then required to produce extracellular Se^0^ nanoparticles in *Rsp. rubrum*.

**Fig. 2:**
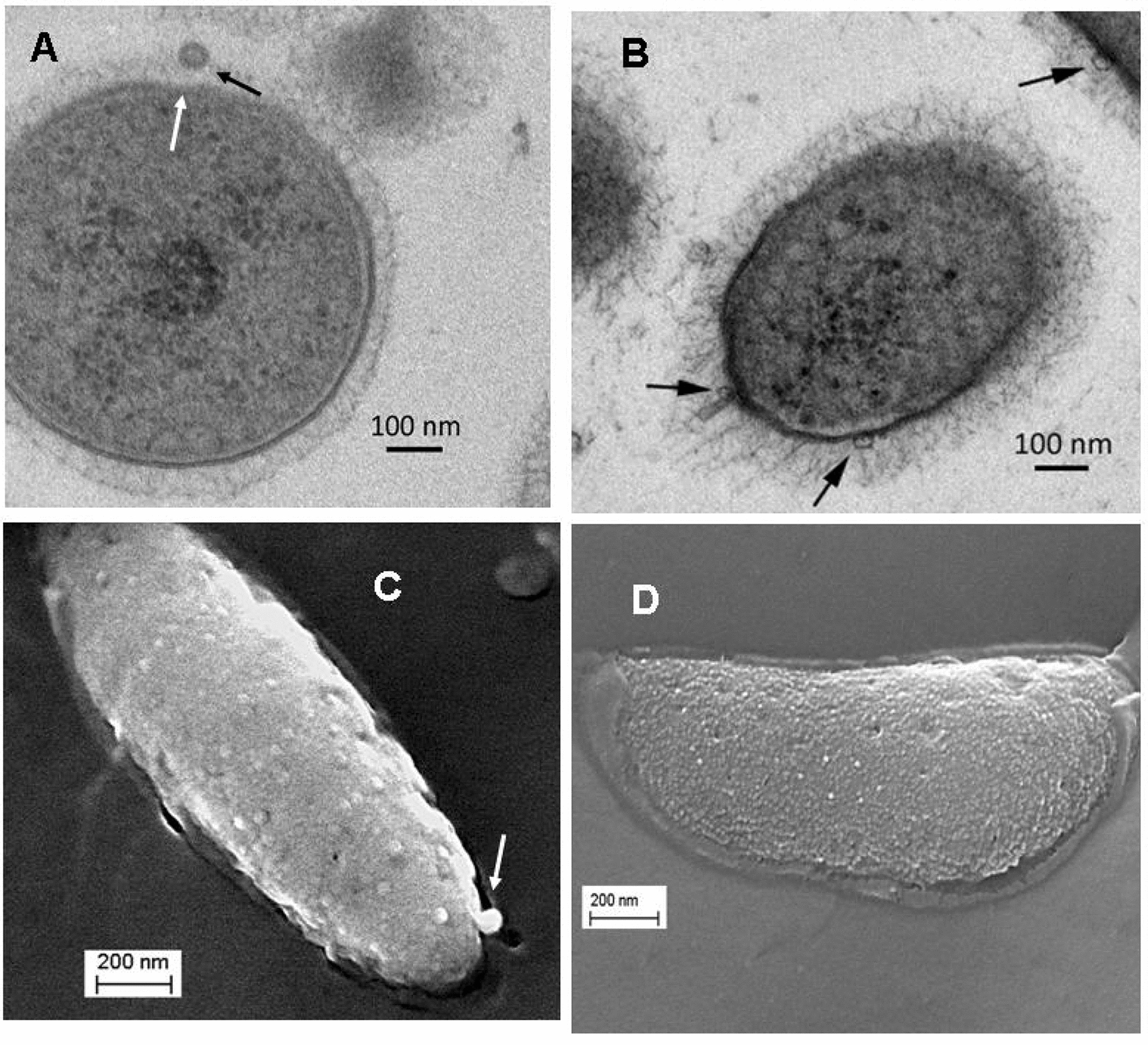
EM micrographs  of *R. capsulatus* in the presence (**A**) or absence (**B**) of selenite, are shown. EM micrographs presented in **A** and **B** were obtained using scanning-electron microscopy (scanning-EM). In** A**, (cell grown in the presence of 0.5 mM selenite), an elemental selenium particle (approx 55 nm size) is still slightly embedded into the OM layer (black arrow) while the cell membrane seems to be slightly modified at the place where the selenium particle is located (white arrow). In **B**, (control cell) extracellular membrane vesicles (EMV) are seen attached to the OM layer of the cells (see black arrows) with diameters varying between 30 and 36 nm . Technical details as in Materials and Methods of Wild et al. [167]. EM micrographs presented in **C** and **D** were obtained using cryo-electron microscopy (cryo-EM). In **C** (cell grown in the presence of 0.5 mM selenite) a particle of about 50 nm, likely containing elemental selenium, is protruding from one of the poles of the cell (white arrow), while on the control cell (**D**), significantly smaller structures are seen on the cell surface. Comparison of this image with that presented in **B** suggests that the small structures present on the cell surface of **D**, likely represent EMV. The small cavities, or membrane alterations seen on the cell surface of **C**, may represent membrane areas damaged by the passage of membrane coated SeNPs (see Additional files 5, 6 and 7 in [120]), and those present on the cell surface of **D**, are proposed to represent membrane areas damaged by the excretion of EMV. (These EM micrographs were obtained in the Center for Microscopy and Image analysis of the University of Zurich, with bacterial cells produced in J Kessi’s laboratory; unpublished material) [56, 158]

Davide: Janine, don't rush forward. The problem related to the presence of Te^0^ and/or Se^0^ nanoparticles outside the cells, either on the surface or simply in the suspension medium or in both sites, is quite complex and must be treated with caution, although the images in Fig. 2 (this work) and those by Yurkov et al. [148], are intriguing. In addition, in your experiments the SeNPs appear outside the cells after several days of growth, as opposed to when using lawsone where the particles are formed from the beginning of growth in the external medium: it doesn't seem like a small difference to me.

Ray: Yes, I agree it’s a complex topic, but to me it reflects the subtle differences between selenite and tellurite recalled by Janine at the beginning of our discussion. Using the same organism and similar growth and exposure conditions, we’ve seen with *R. aetherivorans* BCP1 that tellurite reduction leads to long-rod in the cytoplasm, and they remain there [91, 162]. However, upon selenite exposure, Se^0^ nanospheres and rods were observed extracellularly, apparently associated with the cell surface [163], although I still think the Se atom nuclei were likely produced intracellularly in this Gram-positive strain.

Janine: On this point, however, I want to venture a provocation: in line with my early results [1], I’m convinced that selenite is reduced to SeNPs in the cytoplasm, and that the nanoparticles (in the form of nanospheres) are then extruded into the culture medium as almost no particles were seen in the cytoplasm two days after selenite reduction was completed (shown in [1]). Further, both cell protein measurements and TEM pictures show that most of the cells survived after the extrusion of the SeNPs. In my view, the partial cell death in *Rsp. rubrum* cultures during and after selenite reduction is due to uncontrolled selenite uptake by a fraction of the cell population, leading to accumulation of an excess of SeNPs in the cytoplasm with subsequent cell lysis.

Ray: Let me point out that also for the SeNPs production from *S. maltophilia,* it was proposed that selenite is reduced cytoplasmically through reactions with GSH. The model leads to Se atom agglomeration into NP nuclei, that grow becoming surrounded by amphiphilic compounds, perhaps lipids, and subsequent budding from the membrane leading to SeNPs secretion/excretion via membrane vesicles. The membrane nature around the SeNPs was shown with a lipid dye [159]. This follows Janine’s interpretation as well. However, in the case of *Ochrobactrum sp.* MPV1, the SeNPs either as spheres or rods remained intracellular, regardless of the conditions explored [56], which is different from what I described earlier for *R. aetherivorans* BCP1, were the Se^0^ nanomaterials were found on the cell surface.

Janine: As reported by others [164] membrane permeation of nanoparticles^7^ depends on different properties of the particles, such as size, shape, charge, hydrophobicity, electrophilicity, and most likely on the particular properties of the cell membrane itself. According to this, the membrane permeation occurs when the adhesion energy of the particle with the membrane is sufficient to overcome the energy cost associated with bending the membrane around the cell surface ([164] and references therein).

Davide: In this respect, I’d like to draw your attention to a recent study by Jahan et al. [168] on the role of an outer-membrane porin-like protein, ExtI, in selenite permeation in *Geobacter sulfurreducens*. This work shows that selenite uptake and selenium nanoparticle formation were impaired in an *extI*-deficient strain. Further, the localization on the outer membrane of a putative rhodanese-like lipoprotein, which is encoded by an *extH* gene located immediately upstream of *extI* in the genome, was strongly affected by *extI* deficiency suggesting a direct protein–protein interaction between ExtI and ExtH. This result is intriguing as it suggests a possible relationship between selenite entry through ExtI and its processing by ExtH. What do you think?

Janine: *Geobacter sulfurreducens* belongs to the δ-group of the Proteobacteria and the ExtI is a novel type of porin-like protein, that is unique to the Geobacteraceae family of the δ-proteobacteria. Considering the lack of reports on the presence of this protein in other organisms, it would be a gamble to speculate about its role in the oxyanion processing by genera of the α-group such as *Rhodobacter* and *Pseudomonas*. At present, I strongly support the conclusion made in numerous publications that the reduction of oxyanions occurs mainly in the cytosol and that the particles are eventually transferred outside of the cells.^8^ Supporting this proposition, scanning-EM and cryo-EM pictures of *R. capsulatus* cells presented in Fig. 2, show a SeNP still slightly embedded in the OM (A), or protruding from one of the poles of the cell (C), and membrane vesicles attached to the OM layer of the cell (B). These micrographs also show membrane areas slightly damaged by the excretion of SeNPs (C) and EMV (D).

Ray: I agree that cytosolic processing of selenite to Se^0^ and thus to SeNPs, is most likely the primary process for most bacteria although it is possible that specialized systems to process chalcogen oxyanions differently, could have evolved in extreme niches. However, I am not fully convinced about SeNPs diffusing across the membrane. Indeed, some particles we see are quite large, 10 to 100 times larger than the width of a membrane and as such I would expect loss of membrane integrity. In fact I have started to consider perhaps some of the newly discovered secretion systems such as the new Type 10 SS could be an interesting candidate [171]. Clearly, this specific issue will require some clever experiments to further clarify this process.

Janine: It is right that SeNPs with diameter significantly larger than 50 nm can be seen in the growth medium of selenite-amended cultures after the reduction process to Se^0^ is completed. However, based on my results obtained by ultracentrifugation of cell preparations from *Rsp. rubrum* [1], I am inclined to think that the large particles present in the growth medium derive from small cytosolic particles. For me, it is conceivable that the increase in diameter of the particles occurs after their release from the cells as the size of SeNPs generated outside of *Bacillus mycoides* SeITE01 cells, grown in the presence of selenite, was dependent on the incubation time of the culture with this oxyanion [81]. The same correlation was seen for the extracellular production of TeNPs by cells of *R. capsulatus* in the presence of lawsone [158]. In these publications, the increase in particle size was tentatively explained by an Ostwald ripening mechanism [172–174]. However, I agree with you Ray, that this latter explanation remains in the state of a hypothesis, for the moment.

Davide: Janine, I'm afraid we need to stop our conversation, even though I am sure we could continue with many things left to say. On the other hand, continuing on the latter topic we would risk putting forward too many working hypotheses, leading to greater confusion to those who read this and providing few clear experimental directions.

Ray: Perhaps, after this exciting conversation, we should think about whether the initial question we asked is correct, and whether it is not better to start from the opposite concept, namely: “… how can these two oxyanions be so chemically similar yet so different when they are transformed by bacteria? “

Janine: Joking aside, I guess that even if the initial question was asked in reverse, it would be equally correct. Thus, as proposed by Davide, I stop here without adding anything else, hoping that our discussion will be of some help for further research work on this topic. Thank you again for giving me the opportunity to recall the great contribution that Claudio Vásquez and his research group made to the microbiology and biochemistry of metalloids.

**Fig. 3:**
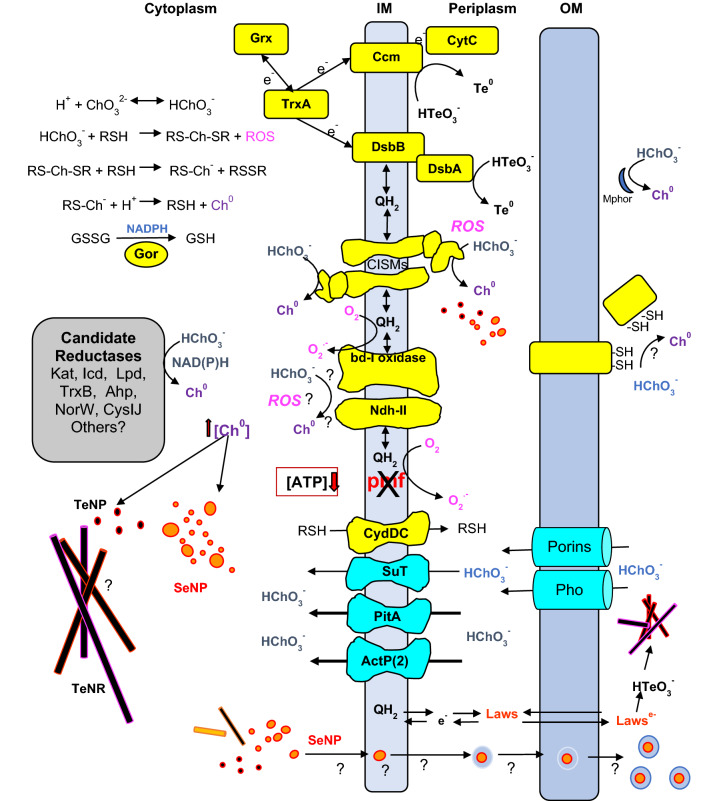
Pictorial overview of the concepts that emerged from the discussion. The cartoon reflects a Gram-negative cell with outer membrane (OM), periplasmic space, and inner membrane (IM), although many of the processes will be the same or similar in Gram-positives. Note: this is a generalized scheme, and the depicted processes may occur differently in different species/strains and growth conditions based on the bioenergetics of their systems. Question marks (?) indicate reactions or biochemical mechanisms not yet clarified, require more experimental support and/or not in agreement. Equations are simplified and are not defined stoichiometrically. See text for further details. Ch, stands for chalcogen metalloid, either selenium or tellurium, unless specifically indicated; GSH, reduced glutathione; CISM, complex iron-sulfur molybdoenzyme; TrxA, thrioredoxin; TrxB, thrioredoxin reductase; Grx, glutaredoxin; Gor, glutathione reductase; ndh-II, NADH:quinone oxidoreductase II; Icd, isocitrate dehydrogenase; Lpd, lipoamide dehydrogenase; Ahp, hydroperoxide reductase; norW, NADH:flavorubredoxin; Ccm, cytochrome C maturation; CydDC, glutathione/cysteine exporter; CysIJ, sulfite reductase; Pit, phosphate importers; SuT, sulfate transporters; Mphor, Metallophore/Siderophore; pmf, proton motive force; QH_2_, reduced quinone; Laws, Lawsone; e-, electrons; GSH, reduced glutathione; GSSG, oxidized glutathione; RSH or -SH, thiol-group containing compounds or amino acids; ROS, reactive oxygen species; NP. Coloration of the nanomaterials: orange or black is elemental Se or Te, respectively; red outline indicates cytoplasmic biomolecular coating; light blue is to suggest a lipid and/or EPS coating; pink coating to the Te rods to suggest a biosurfactant coating

Ray: Okay then, perhaps the cartoon that I sketched during this conversation (Fig. 3) will help to summarize our discussion in which we highlighted both the joys and frustrations of research on the interaction between chalcogen oxyanions and bacteria.

## Brief summary of the three voices discussion

§ 1. In the initial paragraph, the conversation has defined that there are some key physicochemical similarities and differences between the Se and Te oxyanion forms and that there are knowledge gaps in the literature that need to be clarified. Note also, the conversation focuses on the IV redox state i.e., selenite and tellurite, and not the VI state as neither selenate or tellurate are very toxic, nor found at high quantities in the environment.

§ 2. In concluding the second paragraph, the three interlocutors have defined the challenge to understanding metalloid uptake, although recognizing that not everything is known, there is agreement in the following: 1. The oxyanion challenge response is different between bacterial strains regarding the relation of oxyanion uptake to reduction and to the growth profile; 2. Different transporters are used by different bacteria and at different growth states for uptake of the oxyanions into the cytoplasm, including: i) the acetate transporter(s) (ActP) where the oxyanion is likely mimicking the mono-carboxylate group; ii) the phosphate (Pit) and sulfate (SulPT, CysAW) permease systems where the oxyanions mimic the charge and trigonal pyramidal molecular shape. Further, the discussion group recognizes a key knowledge gap of a poor understanding of the genomic response and system regulation response to these oxyanions, which is likely modulating the uptake.

§ 3; 3.1. The discussion later defines that ROS are produced directly by the reaction of selenite/tellurite with SH-groups of cell metabolites, mostly reduced glutathione (GSH), via the Painter reaction, or indirectly through uncoupled segments of the ETC (electron transport chain). But difficulties in the interpretation of experimental results arose by the fact that, despite the high reactivity of tellurite as compared to that of selenite, a lower amount of ROS is measured in cultures of many organisms amended with tellurite but not with selenite. These results gave the question about the phenomenon leading to lower production of ROS in the presence of tellurite. A response that is proposed by considering the thiol peroxidase activity of diaryl ditellurides and diorganyl tellurides [101, 102]. On the other hand, based on the observation that ROS production depends on growth condition of the bacterial cultures, it was concluded that differences in ROS production between cells facing selenite or tellurite, require further investigation.

§ 3.2., 3.3., 3.4. In these discussion sections, it is clear that there is debate with different points of view on the fundamental toxicity reactions and mechanisms. The group agrees on the fact that tellurite can extract electrons from the ETC – mainly at the UQH_2_/DsbB/DsbA level—resulting in cytoplasmic membrane uncoupling, periplasmic generation of Te^0^ and potential ROS production. Further, within the cytoplasm, the oxidation of RSH groups and GSH leads to dysfunction of many key cellular systems. The group recognizes there are multiple membrane and cytoplasmic enzymes that have been shown to have oxyanion reduction activity, but since their concentrations are fractional to that of GSH, it is held that GSH must catalyse the primary chemistry in the cytoplasm for both oxyanions. In Tables 2 and 3, a list of some literature data concerning the interaction between oxyanions and the levels of thiols and reduced glutathione in the cells, as well as the production of ROS, is reported.

**Table 2:** Examples of the reciprocal effects observed following the variation of the cytosolic pool of glutathione and other thiols in the presence of oxyanions (selenite and/or tellurite) in different bacterial species

Bacterial species	Experimental procedures (metalloid)	Results observed	Refs.
Selenite			
*Rsp. rubrum* *R. capsulatus*	*Inhibition of glutathione biosynthesis in cells grown in the presence of selenite	Decrease of selenite reduction rate	[75]
*Stenotrophomonas maltophilia*	*Inhibition of glutathione biosynthesis in cells grown in the presence of selenite	Inhibition of selenite reduction as a function of the inhibitor* amount	[152]
*Stenotrophomonas maltophilia*	Expression of genes involved in glutathione biosynthesis	Induction of glutamate/cysteine ligase and glutathione synthetase	[152]
*Streptomyces* sp. ES2-5	*Inhibition of glutathione biosynthesis in cells grown in the presence of selenite	Decrease of reduced thiols (RSH) following exposure to selenite	[153]
*Achrobactrum* sp. MPV1	*Inhibition of glutathione biosynthesis in cells grown in the presence of selenite	Delay in selenite reduction to Se^0^	[87]
*Achrobactrum* sp. MPV1	Determination of reduced thiols (RSH) in cells exposed to selenite	Decrease of reduced thiols (RSH) following the exposure to selenite	[87]
Tellurite			
*E. coli*	Determination of reduced thiols (RSH) in cells lysates with DTNB as a reagent	Decrease of RSH amount in cells grown in the presence of tellurite	[2]
*E. coli*	HPLC-analysis of low MW compounds from cells grown in the presence of tellurite	Glutathione is the major target of tellurite toxicity	[135]
*Pseudomonas pseudoalcaligenes*	Determination of reduced thiols (RSH) in cell lysates	Decrease of RSH amount in cells grown in the presence of tellurite	[103]
*Geobacter stearothermophilus*	Expression of genes involved in cysteine metabolism (*cysA, cysB, cysC, cysE, cysI, cysM,cysK*) in the presence of tellurite	Induction of the expression of *cys* genes in parallel with decrease of various reduced thiols	[154]

*Glutathione biosynthesis inhibitor, buthionine sulphoximine; DTNB, 5,5′-dithiobis(2-nitrobenzoic acid); HPLC, high-performance liquid chromatography; MW, molecular weight; RSH, reduced thiols; *cys*, cysteine gene. Bacterial species: *E.coli*, *Escherichia coli*; *Rsp. rubrum*, *Rhodospirillum rubrum*; *R. capsulatus*, *Rhodobacter capsulatus*

**Table 3:** Formation of reactive oxygen species (ROS) in various bacterial species when exposed to selenite and tellurite

Bacterial species	Experimental procedures (metalloid)	Results observed	Refs.
Selenite			
*E. coli*	2D-electrophoresis of soluble and membrane fractions from cell extracts	SOD induction but only in cells grown aerobically in the presence of selenite	[89]
*E. coli*	Construction of mutants *(ΔsodA, ΔsodB, ΔtrxA, ΔtrxB, Δgor, ΔgshA*)	Strains lacking either *sod*A or *sod*B were hypersensitive to selenite. Deletions of either *trx*A, *trx*B, *gor*, and *gsh*A had no effect on selenite sensitivity	[89]
*E. coli*	In vivo use of the fluorescent ROS-sensitive probe DCFH-DA after exposure to selenite	Strong increase of fluorescence in cells exposed to selenite	[48]
Tellurite			
*R. capsulatus*	Non-denaturing PAGE of lysates from cells treated with tellurite (or paraquat)	Strong increase of SOD activity in cells treated with tellurite (or paraquat)	[97]
*E. coli, S. epidermidis*	Construction of mutants *(ΔkatG)*	A *kat*G minus mutant was hypersentive to tellurite; expression of *kat* gene of *S.epidermis* in *E.coli* increases in the latter resistance to tellurite	[138]
*E. coli*	Use of the fluorescent ROS-sensitive probe DCFH-DA in extracts of cells exposed to tellurite	Increase of the fluorescence as a function of tellurite concentration	[155]
*E. coli*	DNA fragments amplification of genes (*sodA, sodB, katG, soxS, gapA)*	Increase of *sodA* and *sodB* with a strong induction of *katG* and *soxS* mRNA synthesis in the presence of tellurite	[155]
*Pseudomonas pseudoalcaligenes*	In vivo use of the fluorescent ROS-sensitive probe DCFH-DA after exposure to tellurite	Strong increase of fluorescence in cells exposed to tellurite	[103]

DCFH-DA, dichloro-dihydro-fluorescein diacetate; SOD, superoxide dismutase; PAGE, polyacrylamide gel electrophoresis. Genes: *gapA*, glyceraldeide-3-phosphate dehydrogenase A; *gor*, glutathione reductase; *sodA* and *sodB*, superoxide dismutase A and B; *soxS*, regulatory protein in *E.coli*; *trxA* and *trxB*, thioredoxin reductases. Bacterial species: *E.coli*, *Escherichia coli*, *R. capsulatus*, *Rhodobacter capsulatus*, *R. sphaeroides*, *Rhodobacter sphaeroides*,

*S. epidermidis*, *Staphylococcus epidermidis*

§ 4. In the final paragraph, the group examines some application aspects related to the reduction of oxyanions to metal crystals arranged as nanoparticles (SeNPs and TeNPs), recalling that the addition of the exogenous electron carrier lawsone (a hydroxynaphthoquinone) involves the extracellular reduction of tellurite through its mediation of electrons from the ETC (Electron Transport Chain). Both selenium- and tellurium-nanoparticles (SeNPs and TeNPs) are found to be surrounded by biomolecules, particularly proteins, e.g. porins, and lipids. The group ends their discussion defining that although Se and Te nanomaterials are typically produced intracellularly, the SeNPs can be released from the cytoplasm, perhaps due to size/shape/charge constraints. Clearly, this specific issue requires further research.

## Data Availability

Not applicable.

## Abbreviations

Cd: Cadmium
CISM: Complex iron-sulfur molybdoenzymes
Cyt: Cytochrome
EDX: Electron dispersion X-ray
EPS: Exopolysaccharides
ETC: Electron transport chain
GSH: Glutathione reduced
GSSG: Di-glutathione oxidized
MIC: Minimum inhibitory concentration
NP: Nanoparticle
NR: Nanorod
NAD(P): Nicotinamide adenine dinucleotide (phosphate)
OM: Outer membrane
Q-cycle: Quinone cycle
ROS: Reactive oxygen species
R-SH: Reduced thiol groups
Se: Selenium
Se^0^: Elemental Selenium
SEM: Scanning electron microscopy
SOD: Superoxide dismutase
Te: Tellurium
Te^0^: Elemental Tellurium
TEM: Transmission electron microscopy
UQH_2_: Reduced ubiquinone
UQ: Oxidized ubiquinone
XANES: X-ray absorption near edge structure

## Footnotes

1 Electronegativity of an atom is a measure of the attractiveness of this atom for the bonding pair it forms with other atoms. Thus, a decrease of electronegativity corresponds to a decrease of the attractiveness of this atom for electron bonding pairs, so to lower the ionization energy generating a faster chemical reaction (in: https://sciencenotes.org/list-of-electronegativity-values-of-the-elements/, a periodic table of the elements with indication of their electronegativity is presented). It derives that a strong difference of reactivity between tellurite and selenite is likely, and it may explain the high difference of the minimum inhibitory concentration (MIC) of selenite and tellurite on bacterial cultures, namely: in the micromolar range with tellurite [12, 42–45], while in the millimolar range with selenite [34, 46–48].

2 It has been noted, however, that the monoclinic crystalline form of selenium is metastable under ambient pressure and temperature and to be slowly transformed to the trigonal form. According to Shu et al. [51], the instability of monoclinic selenium crystals is due to an overall heat of transition from the monoclinic selenium crystal form to the trigonal form of − 2.1 kJ/mole Se. These authors also proposed that the main reason for the stability of trigonal selenium is due to its higher packing density. The large difference of crystal structure between monoclinic Se – which is formed of Se_8_ crown ether rings – and trigonal Te – which is formed of infinite helical chains – might explain the difference in particle shape during the reduction of selenite and/or tellurite by bacteria. Indeed, Se_8_-ring crystals possessing similar dimensions on each of their three-dimensional axes, lead to the formation of spherical particles, whereas infinite helical Te-chains contained in its trigonal crystal, lead to the formation of needle-like or rod-shaped crystals and particles.

3 Cultures grown in a Siström medium in the presence of 0.5 mM selenite grew somewhat slower compared with control cultures, but no extended growth lag phase was observed, and the cultures attained a final cell density corresponding to approximately 85% of that of control cultures. In these cultures, selenite was reduced during the exponential growth phase (see Fig. 2 in [75]). In contrast, cultures of *Rsp. rubrum* faced with 0.5 mM selenite developed very slowly at the beginning of growth, and delayed selenite reduction to the end of the exponential phase. Yet in these cultures, the selenite reduction rate was very fast once initiated (see Fig. 1 in [75] and [1]).

4 An interesting research project to be done in the near future should be focused on the mechanisms these bacteria use to sense the presence of selenite and to verify whether these organisms induce various enzymes as those involved in GSH metabolism, in oxidative stress responses [89], in protein translation [46, 89] ahead of selenite reduction, in order to enhance their resistance to this oxyanion. An open question exists: what are the system regulation responses to these oxyanions?

5 We have recently shown that the cyclic voltammogram of tellurite changes in the presence of lawsone, because the two partners interact by shifting the potential of tellurite which is reduced via lawsone [158]. Then, we demonstrated that lawsone moves electrons from the cells and acts as an electronic shuttle between the membrane and tellurite [158]. Put simply, the lawsone shifts the redox balance between the inner/cell membrane and the outer medium preventing tellurite from entering into cells using the acetate (ActP2) and phosphate (PitA) carriers. With lawsone present, a little amount of tellurite still enters, but we estimated that at least 90% is precipitated as Te^0^ needle-like crystals in the culture medium.

6 Recently, R. Borghese and collaborators (unpublished) have compared the size of the TeNPs produced in the w.t. and in single and/or double mutants of the DsbAB/Ccm system (DsbA^−^, DsbB^−^, DsbA^−^/ccdA^−^, DsbB^−^/ccdA^−^) in the presence of lawsone. In single mutants, the TeNPs are much larger than in the w.t., and in double mutants they are even larger than those produced in single mutants. The naivest interpretation of the results is that in w.t. cells of *R. capsulatus* the DsbAB/Ccm system competes with lawsone in the periplasmic reduction of tellurite; conversely, in DsbAB/Ccm mutant cells the lawsone conveys all the available electron pool towards the aggregation nuclei to form Te^0^ crystals (TeNPs) outside of the cell. Presently, we do not know if these aggregation nuclei are on the surface of the external membrane or in the protein-fraction of the cell growth medium.

7 Apart from *Rsp. rubrum*, which we have extensively mentioned [1], excretion of TeNPs or SeNPs by bacterial cells has been proposed in several publications. In *Enterobacter cloacae*, Losi and Frankenberger [165] reported that formation of Se^0^ nanoparticles occurred near the cell surface and was followed by its rapid expulsion outside the cells. In tellurite- and selenite-resistant bacteria from hydrothermal vents, Rathgeber et al. [166] observed accumulation of particles both inside and outside the cells and concluded that the particles were released from the cells. In *Rhodopseudomonas palustris*, Li et al. [85] observed particle accumulation in the cell cytoplasm during selenite reduction, while numerous particles were present in the culture medium after selenite reduction was completed. As cells were not severely damaged in the presence of selenite, they concluded that *R. palustris* was able to efficiently transport the particles out of the cells. Similar results were reported for *S. maltophilia* [82]. These authors reported that selenite reduction activity was detected in the cytoplasmic fraction, while biogenic SeNPs were localized mainly in the extracellular medium. All hypothesize a releasing mechanism from the intracellular environment.

8 Based on the reports of Nakamura and Watano [169] and Verma and Stellacci [170], direct NPs-permeation may occur with particles smaller than 50 nm, namely: i. permeation is observed regardless of the surface charge of the NPs, ii. spherical particles of similar size are taken up 5 times more rapidly compared to rod-shaped particles, and iii. NPs composed of amphiphilic random copolymers exhibit direct permeation, while NPs composed of solely hydrophilic polymers failed to go across the membrane. These results suggest that increase of the NPs surface/volume ratio decreases the membrane permeation rate of the NPs up to a surface/volume ratio where no permeation cannot happen anymore, i.e., when the energy cost associated with bending the membrane around the particles becomes equal to adhesion energy. These rules would explain the difference of permeation between spherical SeNPs and rod-shaped or needle-like TeNPs.
